# Role of the Gut Microbiota in Regulating Non-alcoholic Fatty Liver Disease in Children and Adolescents

**DOI:** 10.3389/fnut.2021.700058

**Published:** 2021-06-25

**Authors:** Daisuke Tokuhara

**Affiliations:** Department of Pediatrics, Osaka City University Graduate School of Medicine, Osaka, Japan

**Keywords:** bile acid, children, dysbiosis, gut microbiota, lipopolysaccharide, non-alcoholic fatty liver disease, short-chain fatty acid

## Abstract

Non-alcoholic fatty liver disease (NAFLD) is the most common form of chronic liver disease in children and adolescents. Although obesity is the leading cause of NAFLD, the etiologies of NAFLD are multifactorial (e.g., high-fat diet, a lack of exercise, gender, maternal obesity, the antibiotic use), and each of these factors leads to dysbiosis of the gut microbiota community. The gut microbiota is a key player in the development and regulation of the gut mucosal immune system as well as the regulation of both NAFLD and obesity. Dysbiosis of the gut microbiota promotes the development of NAFLD *via* alteration of gut-liver homeostasis, including disruption of the gut barrier, portal transport of bacterial endotoxin (lipopolysaccharide) to the liver, altered bile acid profiles, and decreased concentrations of short-chain fatty acids. In terms of prevention and treatment, conventional approaches (e.g., dietary and exercise interventions) against obesity and NAFLD have been confirmed to recover the dysbiosis and dysbiosis-mediated altered metabolism. In addition, increased understanding of the importance of gut microbiota-mediated homeostasis in the prevention of NAFLD suggests the potential effectiveness of gut microbiota-targeted preventive and therapeutic strategies (e.g., probiotics and fecal transplantation) against NAFLD in children and adolescents. This review comprehensively summarizes our current knowledge of the gut microbiota, focusing on its interaction with NAFLD and its potential therapeutic role in obese children and adolescents with this disorder.

## Introduction

**Figure 1:**
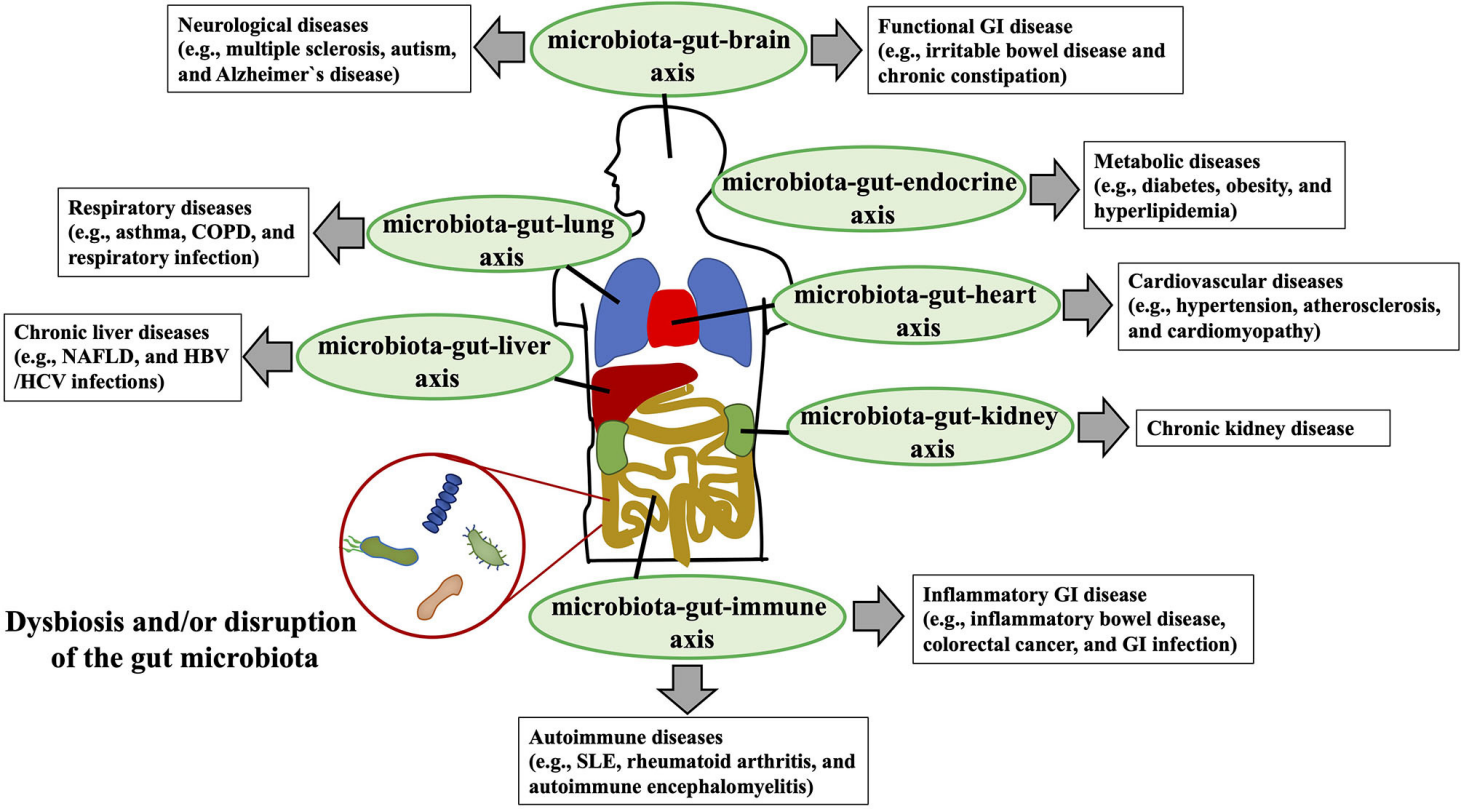
Interaction between dysbiosis or disruption of the gut microbial community and human diseases. Dysbiosis or disruption of the gut microbiota composition that is induced primarily through an imbalanced lifestyle (e.g., high-fat, low-fiber diet) leads to the disruption of the gut barrier, increased gut permeability, systemic circulation of bacterial endotoxins [e.g., lipopolysaccharide (LPS)] to multiple organs (e.g., liver, brain, kidney) and functional systems (e.g., immune system, endocrine systems), and altered production of beneficial metabolites [e.g., short-chain fatty acids (SCFAs)] by normal gut microbiota. Bidirectional and unfavorable crosstalk among the dysbiotic microbiota, gastrointestinal system, and target organs and functional systems (e.g., microbiota-gut-liver axis, microbiota-gut-brain axis) is involved in the pathogenesis of the various diseases (e.g., NAFLD, chronic kidney diseases, Alzheimer's disease, asthma) of the host. COPD, chronic obstructive pulmonary disease; NAFLD, non-alcoholic fatty liver disease; GI, gastrointestinal; SLE, systemic lupus erythematosus; HBV, hepatitis B virus; HCV, hepatitis C virus.

At least 10^14^ diverse micro-organisms, dominated by anaerobic bacteria and representing 500–1,000 different species (1), populate the human gastrointestinal tract (2). In general, these commensal gut microbiota provide beneficial functions for the host, contributing to overall metabolism (e.g., bile acids [BAs]) and the conversion of food into nutrients and energy (e.g., short-chain fatty acids [SCFAs]) (3). In children, other well-understood contributions of the gut microbiota are its roles in the development of gastrointestinal structures (e.g., epithelium, gut-associated lymphoid tissues such as Peyer's patches) and gut mucosal immunity (e.g., induction of secretory IgA and regulatory T cells) (4). In this context, the microbiota-induced gut mucosal immune system protects its host from invasion by infectious pathogens, eliminates harmful non-self antigens, and induces and sustains unresponsiveness to dietary antigens in the phenomenon known as “oral tolerance (4).” Disruption of the gut microbiota in children and adolescents upsets the homeostasis of the gut mucosal immune system, potentially leading to severe gastrointestinal infection, inflammatory bowel disease, and food allergy (4, 5). In addition to its significant involvement in the development and homeostasis of the gastrointestinal mucosal immunity, the gut microbiota is gaining broad attention owing to its dysbiosis or disruption-mediated association with diverse luminal and extraintestinal diseases—including gastrointestinal diseases (e.g., inflammatory bowel diseases, colorectal cancer, gastrointestinal infectious diseases) (6–8), metabolic diseases (e.g., obesity, diabetes, cardiovascular diseases) (9), autoimmune abnormalities (e.g., autoimmune encephalitis) (10), neurological disorders (e.g., autism, Alzheimer's disease) (11–13), and chronic liver diseases (e.g., chronic hepatitis B, non-alcoholic fatty liver disease [NAFLD]) (14, 15)—*via* the bidirectional interactions with the dysbiotic gut microbiota, gastrointestinal system, and other target organs or functional systems (e.g., microbiota–gut–brain, microbiota–gut–liver, microbiota–gut–endocrine axes) (Figure 1).

Resulting from excessive fat accumulation in the liver, NAFLD is a spectrum of liver diseases that ranges from benign steatosis to the hepatic inflammation and fibrosis of non-alcoholic steatohepatitis (NASH) (16). Hepatic fat accumulation can be secondary to genetic or metabolic disorders (e.g., glycogen storage disease, lysosomal acid lipase deficiency and citrin deficiency) (17–19), infections (e.g., hepatitis B virus) (20), the use of steatogenic medications (21), ethanol consumption (22), or malnutrition (23); NAFLD is defined by excluding those factors and is increasingly recognized as a leading cause of chronic liver disease in children and adolescents (24). The detailed mechanism underlying NAFLD is not fully understood; however a “two-hit model” is classically hypothesized, in which the simple steatosis (the first “hit”) progresses to NASH in the presence of a second hit, such as oxidative stress (25). A “multiple-hit” hypothesis has subsequently been proposed in which, in the presence of significant accumulation of fat in hepatocytes and systemic and hepatic insulin resistance, multiple physiological alterations simultaneously lead to an imbalance between the antilipotoxic protection system of the liver and the free-radical production in gut and adipose tissue, resulting in endoplasmic stress, oxidative stress, and hepatocyte apoptosis (26).

Obesity is a major contributor to the development of NAFLD in both children and adults (16, 27–29). The prevalence of NAFLD in the pediatric population is reported to be 2.5–9.6% regardless of obesity but increases to 22.5–44.0% in obese patients (30, 31). Because Western-style and high-fat/carbohydrate/caloric diets are major causes of obesity (32), there is a great demand to elucidate the pathophysiology of NAFLD in the context of obesity and diet, and it is rational to consider the potential role of the gut microbiota—which is in direct contact with and metabolizes the ingested diet—in this regard (33). Recent research advances rapidly uncovered the link between gut microbiota and NAFLD, especially regarding obesity-related and high-fat-diet-induced NAFLD in both adults and children. Dysbiosis or disruption of the typically beneficial gut microbiota promotes the development of NAFLD *via* alteration of gut–liver homeostasis, including dysregulation of the gut barrier, transport of lipopolysaccharide (LPS) to the liver, altered BA profiles, and decreased SCFAs. Conventional approaches to prevention and treatment of obesity and NAFLD (e.g., dietary and exercise interventions) have been clarified to recover microbiota dysbiosis and dysbiosis-mediated altered metabolism. In addition, increased recognition of the importance of gut microbiota-mediated homeostasis in preventing NAFLD has suggested the potential effectiveness of gut microbiota-targeted preventive and therapeutic strategies (e.g., probiotics) for combating NAFLD in children and adolescents. Here I highlight the current understanding regarding the role of the gut microbiota in the pathophysiology of NAFLD, focusing particularly on the children and adolescents, and I discuss the potential application of this knowledge in the preventive and therapeutic approaches for pediatric NAFLD.

## Gut Microbiota in Children and Adolescents

**Figure 2:**
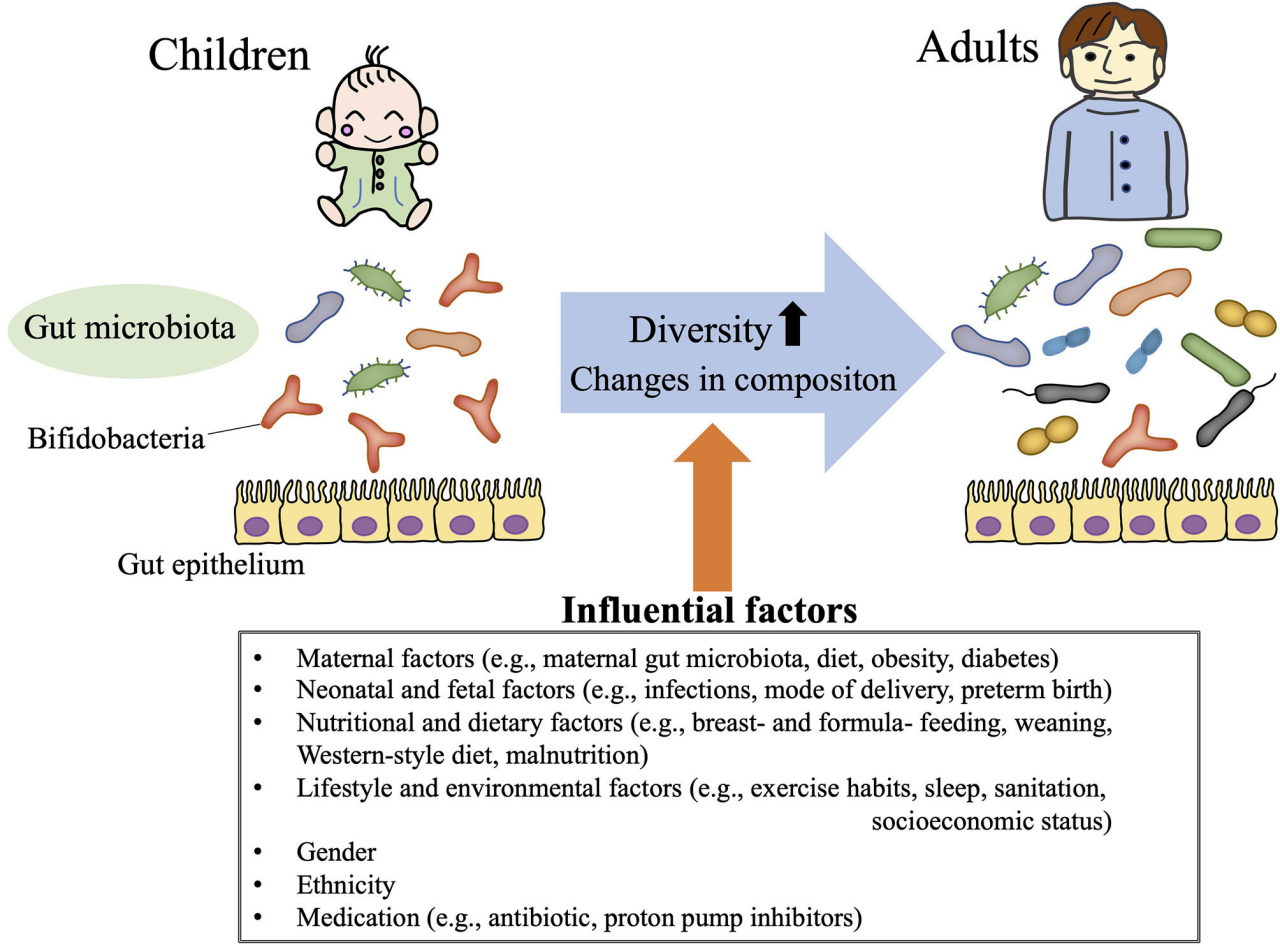
Gut microbiota in children. The gut microbiota demonstrates age-dependent increases in diversity and changes in composition (i.e., relative high abundance of *Bifidobacteria* throughout childhood). These gut microbiota profiles in children are influenced by various life events (e.g., mode of delivery, weaning, and infections), life style choices (e.g., exercise habits, dietary patterns, sleep hygiene), ethnicity, and gender.

In humans, no gut microbiota “signature” (i.e., pattern of component phyla/species) has consistently been attributed to a particular geographic, ethnic, gender, or dietary background (34–37). In addition, the composition of the gut microbiota undergoes age-dependent change, such that microbiota diversity (e.g., richness, evenness) and bacterial abundance differ between even healthy children and adults (38–41) (Figure 2). Neonatal gut microbiomes are colonized with maternal and environmental microbiota and mature toward a stable composition over 2–3 years (38, 42). Distinct microbiome signatures during the neonatal and infant periods are correlated with breastfeeding, formula ingredients, mode of delivery, maternal gestational weight gain, and gender in healthy children (42–45). In addition, weaning itself dramatically alters the gut microbiota composition: a previous study in Japanese children showed that the relative abundance of the phylum Actinobacteria (particularly the genus *Bifidobacterium*) substantially decreased after weaning, such that the phylum Firmicutes became the most predominant (38).

Because the Firmicutes, Bacteroidetes, Actinobacteria, and Proteobacteria are the predominant phyla in human stool samples, most gut microbiota studies of children and adolescents first characterized the abundance of those phyla. The gut microbiota during childhood is less diverse (low abundance and poor richness in bacterial composition) than that of adults (40, 41), gradually increasing in diversity with age (40) (Figure 2). The gut microbial populations of older children are assumed to be similar to those of adults, but the composition of the gut microbiota still differs significantly between adolescents and adults (39). Although no age-associated gut microbiota profile consistently characterizes children from adolescents, a high prevalence of *Bifidobacterium* (within the phylum Actinobacteria) is a well-known signature of for children compared with adults (40) (Figure 2). Bifidobacteria are some of the first species to colonize the newborn gut (46, 47), and adolescents still maintain a higher abundance of *Bifidobacterium* than adults (39). The levels of *Bifidobacterium* decrease gradually throughout childhood and adolescence until stabilizing in early adulthood.

In addition, gut microbiota diversity or composition in children varies according to ethnicity and geographic region or country (48, 49). For example, in a previous study focusing on Asian school children, Bifidobacteriaceae and Bacteroidaceae were abundant in Japan and China, whereas Prevotellaceae was abundant in Indonesia and Thailand (48). Compared with their European counterparts, children (1–6 years of age) in rural Africa, where the diet has a high fiber content, had significant depletion of the phylum Firmicutes and significant enrichment of Bacteroidetes, with unique increases in bacteria from the genera *Prevotella* and *Xylanibacter—*Bacteroidetes members known to carry genes for cellulose and xylan hydrolysis (49). Gut microbiota composition differs by country, suggesting that eco-geographic dietary variations shape the gut microbiota. However, even within the same region (e.g., Latin America), socioeconomic status influences gut microbiota diversity and composition in children (50). Age is positively correlated with increased microbiota diversity in normal-weight children; in contrast, obesity and overweight, which are closely associated with lower socioeconomic status, diminish the maturation of microbial diversity that is associated with age, such that the correlation between age and increased gut diversity is lost in overweight and obese children (50). Similarly, malnutrition—a result of low socioeconomical status, like obesity—leads to gut microbiota immaturity and delays the development of gut diversity in children (51). Therefore, results of the gut microbiota research should be interpreted carefully according to the ethnic, age, dietary, nutritional, geographic, gender, and socioeconomic status of the study population before applying those data to other groups of children and adolescents.

## Gut Microbiota Diversity in NAFLD

Accumulated data clarified that dysbiosis or disruption of the gut microbiota is associated with NAFLD in both children and adults (52–61). The NAFLD-associated gut microbiota typically is described as showing decreased α-diversity (richness and evenness), significantly altered β-diversity, and significant differences in the abundance of bacteria at the phylum, class, family, or genus level, compared with the microbiota of appropriate control subjects.

In adult populations, NAFLD is associated with lower bacterial diversity, increased abundance of the phylum Bacteroidetes, and decreased abundance of the phylum Firmicutes compared with non-NAFLD controls, according to a Taiwanese study (58). In another study, the genus *Ruminococcaceae UCG-010*, family *Ruminococcaceae*, order *Clostridiales*, and class *Clostridia* were all less abundant in patients with non-alcoholic fatty liver or NASH than in healthy controls (58). An increased Bacteroidetes:Firmicutes (B:F) ratio—due to increased abundance of the phylum Bacteroidetes and decreased abundance of Firmicutes—is sometimes reported as a dysbiotic marker characterizing NAFLD (62), but those phylum-level changes are inconsistent. For example, a South Korean study reported decreased abundance of Bacteroidetes and increased abundance of Proteobacteria and Fusobacteria in adult patients with NAFLD compared with healthy adults (53). Therefore, whether an increased B:F ratio should be adopted as the signature of NAFLD-induced dysbiosis remains controversial.

Obesity is a major etiology of NAFLD, and obesity-induced dysbiosis contributes to the development of NAFLD (63). Non-obese adult patients with NAFLD also demonstrate gut dysbiosis, characterized as decreased diversity and phylum-level changes (reduced Firmicutes and increased Bacteroidetes) in gut microbiota composition (63). Furthermore, the gut dysbiotic pattern varies among NAFLD patients depending on their body mass index (BMI) (53). A decrease in Desulfovibrionaceae (phylum Proteobacteria) is associated with NAFLD in lean—but not obese—adults with NAFLD (53). Therefore, gut dysbiosis is associated with NAFLD regardless of obesity or non-obesity, but determining a particular profile (e.g., increased B:F ratio) or specific phyla and genera indicative of the development of NAFLD or NASH development in adults is proving difficult.

As in adults, dysbiosis of the gut microbiota has been found in children and adolescents with NAFLD or NASH worldwide (52, 54, 56, 57, 59, 61) (Table 1). Despite the absence of a consistent signature of dysbiosis, all of the pediatric studies have found decreased α-diversity, distinct differences in β-diversity, or differing abundance of bacteria at the phylum or genus levels (Table 1). As shown in Table 1, decreased α-diversity (richness and evenness), generally evaluated according to the Shannon and Chao 1 diversity indices, might universally be considered the consistent signature of the dysbiosis associated with pediatric NAFLD. In regard to specific bacteria, one study reported that *Faecalibacterium prausnitzii*, the sole known member of the genus *Faecalibacterium* (phylum Firmicutes) and the main butyrate-producing bacteria in the human gut (64), was the only species whose abundance differed between obese children with and without NAFLD (59). The significance of *Faecalibacterium* in NAFLD has likewise been demonstrated in adults: *Faecalibacterium* was significantly less abundant in adults with NAFLD than in non-NAFLD, BMI-, and sex-matched participants (55). As described later, SCFAs (including butyrate) exert protective effects against the development of NAFLD; therefore, a decrease in *Faecalibacterium* may promote the development of NAFLD *via* the loss of SCFAs-mediated protection.

**Table 1:** Alteration of gut microbiota composition in children and adolescents with non-alcoholic fatty liver disease (NAFLD).

**References**	**Country**	**Study population**		**Microbial diversity**		**Microbial abundance (NAFLD relative to control)**	
		**Age (years)**	**Composition**	**α**	**β**	**Phylum**	**Genus/species**
Monga Kravetz et al. (54)	US	13.3 ± 3.2 12.9 ± 2.8	44 obese NAFLD 29 control^*^	↓	NA	↓ Bacteroidetes↓ B:F ratio	↓*Prevotella*, ↓ *Gemmiger* ↓*Oscillospira*
Stanislawski et al. (56)	US	15.1–16.5	8 NAFLD 99 control	↓	Differed	NA	↑*Paraprevotella* ↑*RF32*, ↑ *Sutterella* ↑*Bilophila*, ↓ *Varibaculum* ↓*Oscillospira*
Schwimmer et al. (57)	US	8–17	87 NAFLD 37 control^*^	↓	No difference	↑ Bacteroidetes↑ Proteobacteria↓ Firmicutes	↑*Prevotella copri* (in severe fibrosis case)
Zhu et al. (61)	US	13.6 ± 3.512.7 ± 3.214.4 ± 1.8	22 NASH 25 obese 16 control	↓	Differed	↑ Bacteroidetes↑ Proteobacteria↓ Firmicutes↓ Actinobacteria↑ B:F ratio	↑*Prevotella*, ↑ *Escherichia* ↓*Alistipes*, ↓*Blautia*↓*Coprococcus*, ↓ *Eubacterium* ↓*Oscillospira* ↓*Bifidobacterium*
Del Chierico et al. (52)	Italy	7–16	27 NAFL 26 NASH 8 obese 54 control	↓	Differed	↑ Actinobacteria↓ Bacteroidetes	↑*Dorea*, ↑ *Ruminococcus* ↓*Oscillospira*
Zhao et al. (59)	China	9–17	25 obese + NAFLD 18 obese 15 control	No difference	NA	↑ Proteobacteria↓ Bacteroidetes	↑*Phascolarctobacterium* ↓*Lactobacillus* ↓*Oscillibacter* ↓*Ruminiclostridium*

*↓, decreased in NAFLD or non-alcoholic steatohepatitis (NASH) groups compared with controls; ↑, increased in NAFLD or NASH groups compared with controls; B:F ratio, Bacteroidetes/Firmicutes ratio; NA, not available*.

* *Obese subjects without NAFLD*.

In another study, reduced proportions of the genus *Oscillospira* and high abundance of *Dorea* were significantly associated with NASH in children and adolescents compared with healthy controls (52). The decreased abundance of *Oscillospira* has been confirmed in several studies of NAFLD in children, but *Oscillospira* is just one of the many bacteria that contribute to dysbiosis in NAFLD. In addition, the relative abundance of these NAFLD-specific bacteria varies widely between studies (Table 1), possibly reflecting differences between countries, lifestyle factors, research methods, and so on and thus making it difficult to determine the responsible gut microbiota composition in the development of NAFLD. Considering these findings together, it is safe and rational to say that decreased microbial diversity and disruption of the composition of the normal gut microbiota that is appropriate for the host's geographic region, age, lifestyle, gender, and ethnicity contribute to the development of NAFLD.

Gut dysbiosis is associated not only with the presence of NAFLD but is also related to the disease progression in both NAFLD and NASH. In adults, the pronounced liver fibrosis of NAFLD enhances the increase of the phylum Bacteroidetes and decrease in microbial diversity (60). The gut microbiota profile is further influenced by the presence of hepatocarcinogenesis in NAFLD patients (65). In children and adolescents, a reduced B:F ratio and decreased abundance of *Bacteroidetes, Gemmiger*, and *Oscillospira* are significantly associated with exacerbation of the hepatic fat fraction in obese NAFLD patients (54). Disease progression in NAFLD typically is evaluated through liver biopsy (66), blood biochemical parameters (29, 67), ultrasonography (68), transient elastography (28), magnetic resonance imaging, and elastography (69). Several studies have shown the association of NAFLD with a particular pattern of dysbiosis (53, 56–59, 61–63), and these gut microbiota diversity patterns are applied to estimate the severity of NAFLD (56, 70). Assessment of two taxa (*Bilophila* and *Paraprevotella*), combined with calculating the dietary intake of monounsaturated fatty acids and BMI z-score, was useful to define the hepatic fat fraction in adolescents in the United States (56). A German study demonstrated that changes in gut microbiota diversity can be useful in predicting advanced fibrosis in adult NAFLD patients and that transient elastography achieved the best diagnostic performance regarding the detection of NAFLD patients at risk for disease progression (70). Furthermore, compared with those with mild fibrosis, patients with significant liver fibrosis had a higher abundance of the genera *Escherichia* and *Shigella* and the corresponding family Enterobacteriaceae (53). As mentioned previously, these findings should be interpreted carefully, because detailed gut microbiota profiles differ among NAFLD patients and controls with differing background (e.g., country, age, gender) populations. However, the accumulation of gut microbiota data is expected to evolve into a new non-invasive method for estimating the severity of NAFLD in target populations.

## Mechanisms of Dysbiosis-Induced NAFLD

**Figure 3:**
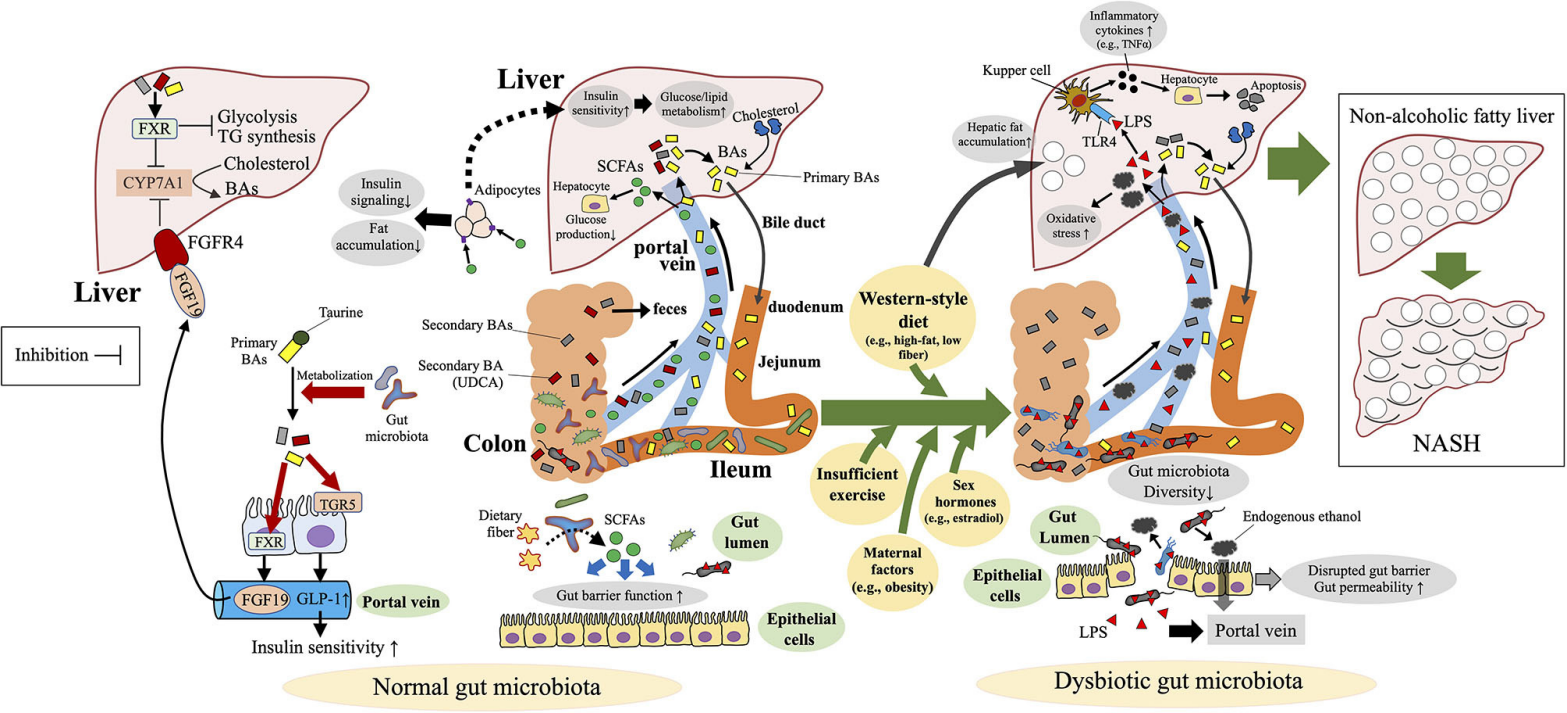
Mechanisms of dysbiosis-induced NAFLD in children and adolescents. Dysbiosis and disruption of the gut microbiota contribute to the development of non-alcoholic fatty liver disease (NAFLD) *via* modulation of the gut–liver homeostasis, including the involvement of the gut barrier, bacterial endotoxin [lipopolysaccharide (LPS)], endogenous ethanol, bile acids (BAs), and short-chain fatty acids (SCFAs). FGFR4, fibroblast growth factor receptor 4; FXR, farnesoid X receptor; TGR5, G-protein-coupled bile acid receptor; TLR4, toll-like receptor 4; TNF-α, tumor necrosis factor-α; UDCA, ursodeoxycholic acid.

Dysbiosis or disruption of the gut microbiota is not only associated with NAFLD but also has an etiologic role in the development of NAFLD *via* modulation of the gut–liver homeostasis, including the involvement of the gut barrier, LPS, BAs, and SCFAs (Figure 3). Animal studies using fecal transplantation or antibiotic treatment have clearly indicated the pathologic role of a disrupted gut microbiota in the development of NAFLD (71–74). Whereas, germ-free mice are protected against high-fat-diet-induced obesity (75), the transplantation of feces from NASH patients enhanced high-fat-diet-induced hepatic steatosis and inflammation in the recipient mice (71, 72). In another study, antibiotic treatment at sub-therapeutic doses induced an immature microbial community and led to the obesity phenotype and NAFLD in adult mice fed a high-fat diet (74). In a mouse pediatric model, antibiotic exposure soon after birth disrupted the gut microbiota and enhanced the effect of high-fat-diet-induced obesity (73). Transplantation of the gut microbiota raised after the antibody treatment induced obesity and NAFLD in recipient germ-free mice, thus clarifying that the altered microbiota—not the antibiotics themselves—plays a causal role in the development of NAFLD (73).

Lifestyle factors, including diet, are the most important cause of the dysbiosis and dysbiosis-induced deterioration of the beneficial functions of the gut microbiota in NAFLD (76–80). In mice, a high-fat diet causes obesity, hyperglycemia, and shifts in the diversity of the dominant gut bacteria (e.g., decreased *Ruminococcaceae*, increased relative abundance of *Rikenellaceae* in mice) (77). In another study, a high-fat diet induced gut dysbiosis (decreases in the phlyum Bacteroidetes and increases in Firmicutes and Proteobacteria) independent of obesity (76). Changes in gut microbiome composition primarily begin with variations in the genera *Bacteroides* and *Prevotella* within 24 h of initiating a high-fat/low-fiber diet; in contrast, changes in enterotypes occur with long-term feeding of these diets (78). In addition to the alterations in gut microbial diversity, a high-fat diet modulates the function (e.g., amino acid metabolism) of gut microbiota and cecal metabolic pathways (e.g., BA and bilirubin metabolism) (77). Interestingly, control-diet intervention reverses these high-fat-induced changes in the diversity and function of the gut microbiota (76, 78).

In addition to dietary composition and duration, feeding patterns affect the gut microbiota composition. In juvenile mice, an irregular dietary pattern (time-restricted feeding) induces dysmetabolism (high body weight and blood glucose levels) *via* dysbiosis of the gut microbiota associated with reduced α-diversity (79). In addition, insufficient exercise decreases gut microbiota diversity and microbiota-derived SCFAs in humans, independent of diet (80). Lifestyle-induced disruption of the gut microbiota is further enhanced by antibiotic use during childhood (73).

Accumulated studies are gradually disclosing the mechanisms through which disruption of the gut microbiota contributes to the development of NAFLD. In a murine NAFLD model, dysbiosis of the gut microbiota leads to disruption of the gut barrier and thus exerts the toxic effects of inflammatory cytokines (e.g., TNF-α), components (e.g., LPS), and metabolites (e.g., ethanol) derived from dysbiotic gut microbiota are exerted on the liver *via* the portal vein after intestinal absorption (81–83) (Figure 3). Transmission electron microscopy revealed irregularly arranged microvilli and widened tight junctions in the gut mucosa of adult NAFLD patients (82). In mice, a high-fat diet induces a diet-driven dysbiosis that promotes gut-vascular barrier damage and bacterial translocation into the liver (83). In a murine NASH model, bacteria-derived LPS induces hepatic TNF-α production, consequently stimulating hepatocyte apoptosis (84) (Figure 3). Furthermore, serum levels of LPS-binding protein were elevated in obese human NAFLD patients, among whom hepatic expression of TNF-α mRNA was significantly enhanced in those with NASH (85). Mechanical disruption of the intestinal barrier in mice by using oral dextran sulfate sodium increased portal LPS absorption, hepatic inflammation, and fibrogenesis in the context of high-fat-diet-induced steatosis (86). LPS mediates inflammatory signals (e.g., TNF-α) *via* toll-like receptor (TLR) 4 (87). Plasma LPS concentrations and hepatocyte TLR4 expression are higher in patients with NASH than in those with non-alcoholic fatty liver (simple steatosis) (88).

In addition to LPS, endogenous ethanol, a metabolite of various gut microbiota species, is also suggested to be involved in the pathomechanisms of NAFLD. Increased ethanol levels have been detected in children with NASH but not alcohol consumption (61) as well as in obese adults (89). In children with NASH, disruption of the gut microbiota is associated with abundant alcohol-producing bacteria (*Escherichia*) and elevated blood ethanol concentrations (61). After its absorption, endogenous ethanol reaches the liver *via* the portal vein and causes oxidative stress secondary to hepatic inflammation (Figure 3). These results indicate that inflammation of the intestinal mucosa (mediated through a disrupted gut microbiota), portal transport of LPS and ethanol derived from dysbiotic bacteria, LPS–TLR4 signaling, and ethanol-mediated hepatic inflammation are all involved in the pathogenesis of NAFLD.

SCFAs (e.g., butyrate, propionate, acetate) are key metabolites in the gut microbiota that protect against NAFLD. These metabolites are produced by the gut microbiota in the large intestine through anaerobic fermentation of indigestible polysaccharides, such as dietary fiber and resistant starch (90) (Figure 3). SCFAs are important energy sources for the host (e.g., colonocytes), and evidence showing the significant association of dysbiosis-induced decreases in SCFAs with NAFLD is accumulating (63, 91). In a spontaneous mouse model, the development of metabolic syndrome was associated with the dysbiosis and decreased plasma SCFA levels (91). In addition, adult NAFLD patients and high-fat-diet-induced NAFLD mice both showed decreased numbers of SCFA-producing gut microbiota (63, 92). In this regard, a high abundance of the phylum Proteobacteria and a low abundance of Bacteroidetes in cirrhotic adults leads to their low capacity to ferment non-digestible carbohydrates into SCFAs (93).

Direct evidence of the protective role of SCFAs against NAFLD (94–96) includes a previous study in which feeding of the SCFA acetate to mice on a high-fat diet decreased hepatic lipid accumulation, improved hepatic function, and increased liver mitochondrial efficiency (94). In addition, mice deficient in the SCFA receptor GPR43 become obese on a normal diet, whereas those overexpressing GPR43 specifically in adipose tissue remain lean even when fed a high-fat diet (95). SCFA-mediated activation of GPR43 suppresses insulin signaling in adipocytes, thus inhibiting fat accumulation as adipose tissue and promoting the metabolism of unincorporated lipids and glucose in other tissues (95) (Figure 3).

Among SCFAs, propionate inhibits hepatocyte glucose production by reducing the gene expression of gluconeogenic enzymes independent of insulin signaling *via* a h pathway (97). Furthermore, dietary SCFA supplementation reduced high-fat-diet-induced metabolic abnormalities including NAFLD in mice in an adipose and hepatic peroxisome proliferator–activated receptor γ-dependent manner (96). In addition, SCFAs regulate intestinal barrier function and therefore contribute to the maintenance of intestinal mucosal homeostasis (98) (Figure 3): in particular, butyrate decreases the tight junction permeability of the colon epithelium (99), thus perhaps protecting against dysbiosis-induced gut barrier disruption and portal transport of LPS. Together, these results clearly show that loss of SCFA-mediated protection against hepatic fat deposition and gut barrier disruption are underlying mechanisms of dysbiosis-induced NAFLD.

Other key factors in the interaction between gut microbiota and NAFLD are BAs and the molecules (e.g., farnesoid X receptor [FXR]) that regulate BA synthesis. Primary BAs (e.g., cholic acid, chenodeoxycholic acid) are synthesized from cholesterol in the liver, then taurine- or glycine-conjugated BAs are passed into the duodenum and used for fat absorption *via* the micelle formation from dietary lipids (100) (Figure 3). Remaining conjugated BAs are unconjugated and/or further metabolized into secondary BAs (e.g., deoxycholic acid, lithocholic acid) in the terminal ileum and the colon by the gut microbiota (101). BAs are reabsorbed mainly in the ileum, where they enter the enterohepatic circulation *via* the portal vein and are returned to the liver (102) (Figure 3). When reabsorbed, BAs play key roles in the maintenance of homeostasis of hepatic lipogenesis (e.g., inhibition of triglyceride biosynthesis) and glucogenesis (e.g., increase in insulin sensitivity) (103) through two major receptor pathways: FXR, a nuclear hormone receptor (104), and TGR5, a G-protein-coupled bile acid receptor (105) (Figure 3). Fibroblast growth factor (FGF) 19, is secreted in the small intestine *via* an intestinal FXR-mediated mechanism, circulates to the liver, where it binds to and activates FGF receptor 4, thus repressing the BA-synthetic enzyme CYP7A1 and consequently BA synthesis (106) (Figure 3).

Because BAs are critical regulators of fat absorption and hepatic lipid and glucose metabolism, it is rational to consider that BA and BA-related molecules (e.g., FXR, TGR5, FGF19) contribute to microbiota-associated protection against NAFLD. However, BAs have well-known paradoxical effects—liver toxicity (e.g., disruption of cell membrane, oxidative stress, apoptosis) and liver protection (107) —such that one cannot simply conclude that BAs are solely beneficial for NAFLD patients. Instead, BA homeostasis (i.e., a balanced BA profile) is considered beneficial for the host, and the disruption of BA homeostasis, which is influenced by gut dysbiosis, promotes NAFLD (Figure 3). In fact, NAFLD is associated with altered BA profiles and gut microbiota composition (108–110) (Figure 3).

In children with NAFLD, serum concentrations of primary and secondary BAs and intestinal secondary BA-producing bacteria are increased, whereas hepatic FXR-mediated and FGF receptor 4-mediated BA signaling is suppressed (108). However, another study in children reported decreased serum BAs in early NAFLD and their increase as fibrosis increased (109). In mice, the transplantation of cirrhosis-associated dysbiotic gut microbiota decreased secondary BAs in the intestine and impaired the intestinal barrier (111).

Because BAs are metabolized into secondary BAs *via* the gut microbiota, antibiotic-induced modulation of gut microbiota composition influences BA metabolism (110, 112). For example, short-term antibiotic treatment of mice decreased secondary BA-producing bacteria (e.g., *Bacteroides* species), thus reducing secondary BA levels in the liver and increasing those of hepatic proteins involved in the cholesterol biosynthetic pathway, which is related to the development of NAFLD (110). In mice, two antibiotic combinations (vancomycin+imipenem and cephalothin+neomycin) were shown to increase the intestinal B:F ratio and reduce secondary BAs levels in the serum, liver, and intestine in mice (112). These short-term antibiotic-induced dysbiotic and negative metabolic effects were reversed through dietary supplementation with secondary BAs (110). This finding may explain, from the aspect of a BA-mediated mechanism, why antibiotic treatment promotes the development of high-fat-diet-induced NAFLD *via* the disruption of gut microbiota (73, 74).

A high-fat diet diversely affects BA profiles and the gut microbiota. For example, a high-fat diet induces greater BA production than does either a high-protein or high-carbohydrate diet in both rats (113) and humans (114). In a pediatric animal model, feeding a high-fat diet induced NASH in juvenile pigs that was associated with gut dysbiosis, increased levels of secondary BAs in the colon, and the impaired enterohepatic FXR–FGF19 signaling (115). The administration of BAs (e.g., ursodeoxycholic acid, chenodeoxycholic acid) ameliorates hepatic fat accumulation in high-fat–diet-fed mice (116, 117). A dual FXR–TGR5 agonist decreased the histologic features of NASH in an obese mouse model (118); therefore, BA-induced amelioration of high-fat–induced NAFLD is considered to be mediated *via* FXR and TGR5. Conversely, deoxycholic acid supplementation induced disruption of the gut barrier and gut dysbiosis (119). Taken together, these findings show how BA profiles and BA synthesis are variously altered under the dysbiotic conditions according to the etiology (e.g., antibiotic use, high-fat diet) of NAFLD. It is currently difficult to define in detail particular BA profiles and serum, hepatic, and intestinal BA levels that are applicable to all types of NAFLD. However, regardless of the actual BA profile and its synthetic pathway, the disruption of BA homeostasis is considered to be central to gut dysbiosis-mediated NAFLD in children and adolescents.

## Gender-Associated NAFLD and the Involvement of the Gut Microbiota

Gender-associated differences in the prevalence and severity of NAFLD are well-recognized. In adolescents, NAFLD is generally more prevalent in males than in females (29, 120–122). Among obese US adolescents, boys were 6 times more likely than girls to have elevated alanine aminotransferase (ALT) concentrations (121). In Australia, a small-scale study showed that whereas the prevalence of NAFLD was significantly higher in females than in males, the NAFLD in males was associated with more adverse metabolic features and greater visceral adiposity (123). These gender-associated differences implicate the involvement of sex hormones (e.g., estrogen) in the development of NAFLD in adolescents (124). In particular, estrogen (e.g., estradiol, esterone) is required for the normal development and functioning of the female reproductive system as well as various non-reproductive organ pathologies, including brain damage and liver diseases; estrogen also influences gut microbiota diversity (124–130). In terms of the NAFLD, maintaining an appropriate estrogen level protects against NAFLD-related events. For example, estradiol treatment in rats decreases CCL4-induced liver fibrosis (129). In addition, an increased serum estradiol concentration was associated with lower-grade portal inflammation in girls with NAFLD (124). In another study, 17β-estradiol attenuated weight gain in ovariectomized *ob*/*ob* mice fed a high-fat diet (131). In regard to the gut microbiota, men with high serum testosterone levels or women with increased serum estradiol harbored more diverse gut microbial communities than populations having lower concentrations of these hormones (127). In female mice, estrogen prevents autoimmune encephalomyelitis–associated changes in the gut microbiota and promotes bacterial enrichment (130). In another study, polycystic ovary syndrome in women induced elevated testosterone levels, which correlated with the disruption of the gut microbiota (128). The mechanisms of the gender-associated NAFLD is still unclear, but the close relationships between sex hormones and the development of NAFLD and effects on gut microbiota diversity imply that sex hormone-mediated alteration of the gut microbiota may contribute to the development of NAFLD in adolescents.

## Maternal Influences on the Gut Microbiota and the Development of NAFLD in Their Offspring

A mother's obesity status, pregnancy diabetes, and infant feeding style all affect her offspring's innate immune system and gut microbiota (42, 63, 132, 133) and NAFLD development (133–135). Germ-free mice colonized with stool microbes from 2-week-old human infants born to obese mothers demonstrated increased hepatic expression of genes associated with endoplasmic reticulum stress and innate immunity, together with periportal inflammation, a histological pattern commonly noted in pediatric cases of NAFLD (133). Another study demonstrated that maternal consumption of a Western-style diet induced early gut dysbiosis (i.e., low abundance of *Parabacteroides* and *Lactobacillus*, increased *Ruminococcus*), disrupted intestinal tight junctions, and accelerated liver fibrosis in their offspring, resulting in the polarization of bone marrow-derived macrophages and induction of proinflammatory and profibrotic programs–effects that persisted into adulthood (134).

Although a matter of on-going debate, a mother's milk-feeding style is suggested to influence the development of NAFLD in her offspring (135, 136). According to one observational study, breast feeding reduced children's risk of developing NASH even as late as 3–18 years later (135). The detailed mechanism underlying this fact is not fully understood; however, infant gut microbiota diversity is closely related to breast milk feeding and milk bacterial communities (137). Breast milk bacterial diversity was lower in obese mothers than in non-obese mothers (138). Those findings implicate the critical role of maternal obesity–associated infant dysbiosis in the development of NAFLD in children.

## Conventional Therapeutic Approach to NAFLD and the Interaction With Gut Microbiota

Currently, there is no proven treatment for NAFLD in obese pediatric patients, but weight reduction based on diet changes and increased exercise are generally the primary NAFLD treatment approaches in obese children as well as adults (139, 140). Diet- and exercise intervention–based reduction of body weight are effective in reducing hepatic fat deposition, as measured by transient elastography, in obese NAFLD children and adolescents (141). Although the mechanism through which diet and exercise alter the gut microbiota to ameliorate NAFLD is unclear, appropriate diet and exercise are unequivocally beneficial in the maintenance of a healthy gut microbiota. In terms of exercise, gut microbiota diversity is greater in athletes than in non-athletic healthy persons (142), and aerobic exercise itself—independent of diet—increases fecal concentrations of SCFAs *via* alteration of gut microbiota diversity in human adults (80). In mice, exercise prevents the disruption of the gut microbiota induced by an environmental toxicant (143). Furthermore, 12 weeks of exercise training favorably altered the deleterious obesity-related microbiota profile and reduced microbial inflammatory signaling in obese children (144). Given that the consumption of a high-fat diet—a conventional method for inducing experimental NAFLD in mice—is associated with disruption of the gut microbiota (76), providing a diet that counters the development of NAFLD is a rational therapeutic means for ameliorating a disrupted gut microbiota. In fact, dietary intervention in the form of an energy-restricted, high-protein diet increased the gene richness of the gut microbiome and clinical phenotypes in obese adults (145). In another study, whey protein isolate specifically normalized energy intake, decreased fat mass, and improved the composition of gut microbiota associated with prolonged high-fat feeding (146). Dietary intervention comprising a diet rich in non-digestible carbohydrates induced significant weight loss and modification of the dysbiotic gut microbiota profiles in Chinese obese children with Prader–Willi syndrome or simple diet-induced simple obesity (147). Together, these findings implicate the significant role of the gut microbiota in the dietary– and exercise–mediated amelioration of NAFLD in children and adolescents.

In addition to dietary and exercise intervention, vitamin E supplementation is well-recognized as an anti-NAFLD therapeutic nutrient in both adults and children (148–150). Meta-analysis demonstrated that vitamin E was effective in ameliorating the biochemical and histological characteristics of adult patients with NAFLD, especially NASH (150). In children with NASH, a double-blind, placebo-controlled, randomized trial found that vitamin E treatment did not reduce ALT levels but significantly improved histologic outcomes (148). In another pediatric study, treatment with hydroxytyrosol and vitamin E reduced the NAFLD-related systemic inflammation, mainly *via* increasing circulating IL-10 levels that occurred in response to DNA damage recovery, ultimately decreasing steatosis and hypertriglyceridemia (149).

Direct evidence indicating that vitamin E supplementation ameliorates NAFLD *via* the reduction of gut microbiota dysbiosis is unavailable; however, multiple studies demonstrate the beneficial effects of vitamin E supplementation on the gut microbiota (151, 152). In the dextran sulfate sodium-induced colitis murine model, vitamin E supplementation preserved intestinal barrier function and caused favorable changes in the gut microbiota, which was disrupted by colitis (151). Disruption of the intestinal barrier and increased portal LPS derived from the dysbiotic gut microbiota promote the development of NAFLD (81, 82); therefore, vitamin E effects on improving the intestinal barrier and gut microbiota diversity may implicate gut microbiota-mediated mechanisms in the vitamin E supplementation-induced amelioration of NAFLD (151).

## Gut Microbiota-Targeted Preventives and Treatments for NAFLD

**Figure 4:**
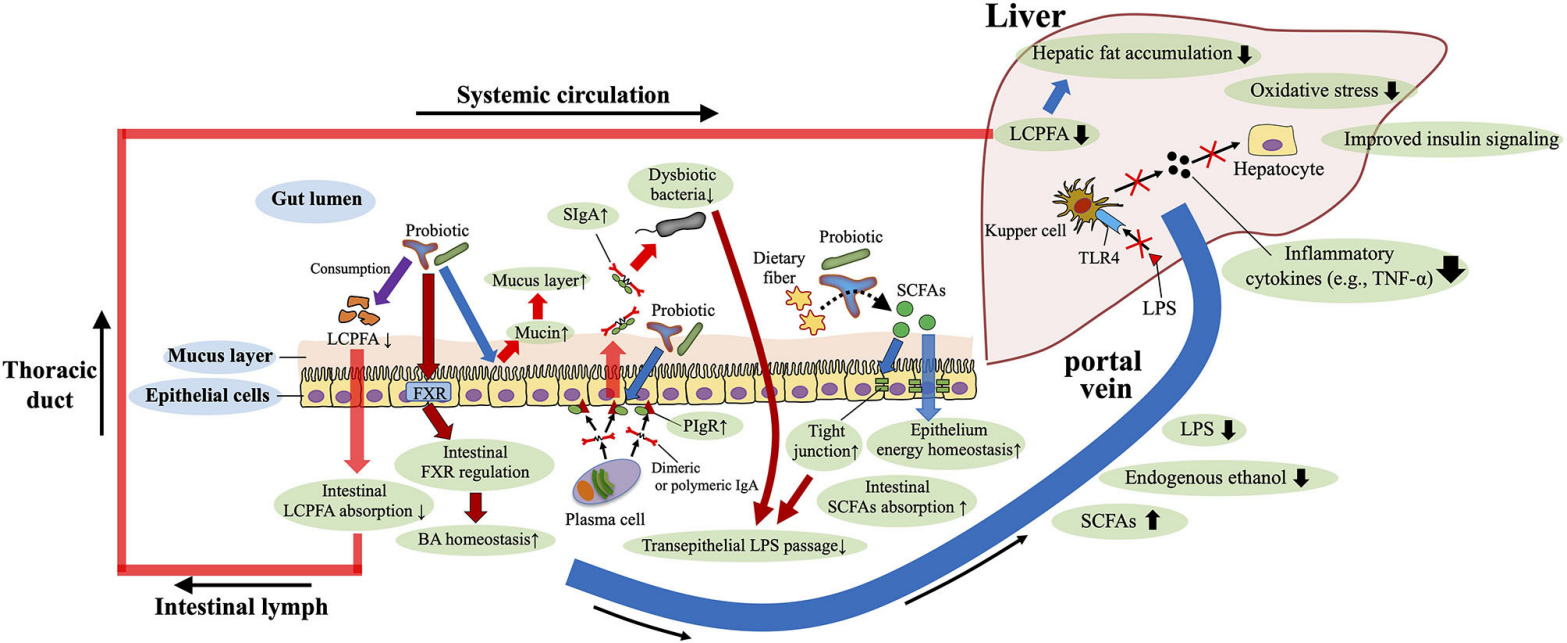
Roles of probiotics against NAFLD. Probiotics enhance the barrier function of the gut [e.g., mucus layer, secretory IgA (SIgA) levels and tight junction tension], and improve the gut microbiota composition, bile acid (BA) homeostasis, and short-chain fatty acids (SCFAs) production. Restored gut barrier function and gut microbiota reduce portal transport of lipopolysaccharide (LPS), and therefore decrease LPS–toll-like receptor 4(TLR4) signaling-mediated inflammatory cytokine [e.g., tumor necrosis factor-α (TNF-α)] production in the liver. Probiotics also reduce intestinal absorption of long-chain polyunsaturated fatty acid (LCPFA) by consuming intestinal LCPFA. These beneficial roles of probiotics are involved in the underlying mechanisms of protection against NAFLD. PIgR, polymeric Ig recetor.

Because gut dysbiosis is involved in the pathomechanisms of the NAFLD, it is rational to consider interventions that target the gut microbiota (e.g., the use of probiotics and fecal transplantation) to treat and prevent NAFLD. Accumulated studies in mice and humans support the beneficial role of probiotic supplementation to protect against NAFLD (81, 153, 154) (Figure 4). In terms of the anti-fibrotic effects of probiotics, *Lactobacillus rhamnosu*s GG supplementation prevents excessive BA-induced liver injury and fibrosis in bile duct–ligated mice by increasing intestinal farnesoid X receptor–mediated suppression of BA synthesis and enhancing BA excretion (155). In addition, supplementation with *Lactobacillus rhamnosus* GG protected mice from high-fructose-diet-induced NAFLD by increasing beneficial bacteria, restoring gut barrier function, and reducing portal transport of LPS (81) (Figure 4). A recent study disclosed that oral administration of *Lactobacillus rhamnosus* GG reduces intestinal fatty acid absorption by consuming intestinal fatty acids, thereby inhibiting the development of NAFLD in high-fat-diet-fed mice (156) (Figure 4). Regarding probiotic-mediated enhancement of the gut barrier, providing mice with IgA-coated *Lactobacillus jensenii* (compared with supplemetation with IgA-free bacteria) effectively inhibited high-fat-diet-induced gut mucosal barrier damage and dyslipidemia. In addition, treatment with IgA-coated *Lactobacillus jensenii* up-regulated mucin-2, polymeric Ig receptor mRNA expression, and colonic butyrate production, all of which serve to enhance the barrier function of the gut (e.g., mucus layer, secretory IgA levels and tight junction tension) (157) (Figure 4). However, to obtain probiotic benefits, the probiotic strain appropriate for the desired purpose must be selected.

Probiotics may improve the gut microbiota structure, reduce liver pathology, and downregulate serum LPS and liver TLR4 levels; therefore, these supplements may delay the progression of NAFLD by decreasing LPS–TLR4 signaling (158) (Figure 4). In human adults with both type 2 diabetes and NAFLD, 8 weeks of administration of a product containing 14 strains of probiotic bacteria significantly improved fatty liver indexes, aminotransferase activity, and inflammatory cytokine levels (TNF-α and IL-6) (153). In addition to the suppression of pro-inflammatory signals, supplementation of the probiotic mixture (e.g., Lactobacillus acidophilus, Lactobacillus plantarum, and Bifidobacterium bifidum) was shown to moderate high-fat– and high-sucrose–diet-induced steatosis *via* the reduction of serum adipocyte hormones (leptin and resistin) in rats (159). In obese Irani children, 12 weeks of treatment with a probiotic product containing *Lactobacillus acidophilus, Bifidobacterium lactis, Bifidobacterium bifidum*, and *Lactobacillus rhamnosus* ameliorated several biochemical parameters (e.g., aspartate aminotransferase, ALT, triglycerides) and hepatic ultrasonographic findings (154). In contrast, another study found no significant effect of probiotic supplementation in obese Latino adolescents; instead, the probiotic increased obesity with no significant changes in the gut microbiota or liver fat and fibrosis (160). These findings support the potential usefulness of probiotics in pediatric NAFLD but also indicate the need to select an appropriate bacterial strain to obtain the favorable effects.

Because fecal microbiota transplantation of dysbiotic microbiota induces the development of the NAFLD phenotype in mice, it is rational to consider the fecal transplantation of healthy gut microbiota as another therapeutic option for NAFLD or NASH, and recent studies support the usefulness of fecal transplantation in ameliorating NAFLD in mice and humans (161–163). In a murine model, 8 weeks of transplantation of a fecal suspension which was collected from healthy mice corrected the gut microbiota imbalance and reduced high-fat–diet-induced NASH, as indicated by a reduction in intrahepatic lipid accumulation and intrahepatic pro-inflammatory cytokines (161). Another murine study in high-fat–diet-fed mice has demonstrated that autologous fecal transplantation (transplantation of the animal's own feces that were collected before disease development) significantly increased bacterial richness and diversity and enhanced the effect of energy restriction on weight loss and adiposity, possibly by decreasing feed efficiency and increasing adipose tissue lipolysis and hepatic fatty acid oxidation (162). In humans, a double-blind randomized controlled study disclosed that allogenic fecal transplantation of feces from lean vegan donors to obese patients with steatohepatitis improved intestinal microbiota composition, plasma metabolites, and markers of steatohepatitis (163). The underlying mechanism of fecal transplantation-mediated amelioration of NAFLD is not fully understood. However, in previous studies of non-NAFLD, fecal transplantation recovered SCFA and BA metabolism in patients with recurrent *Clostridium difficile* infection (164), and improved the injured gut barrier function in *Escherichia coli* K88-infected piglets (165). The beneficial role of fecal transplantation in NAFLD similarly may rest on the recovery of the impaired metabolism (SCFAs and BA) and gut barrier function, both of which are important possible mechanisms explaining dysbiosis-induced NAFLD.

Several studies have examined the anti-NAFLD effects of herbal medicines and functional foods from the aspect of modulation of the gut microbiota and its metabolites (92, 166, 167). For example, ginsenosides—saponins that are the major pharmacologically active components of ginseng root— alle*via*te NAFLD in high-fat–diet-fed mice *via* the modulation of the gut microbiota, consequently enhancing gut barrier function and restoring the energy balance (167). Similarly, supplementing high-fat–diet-fed mice with pectin, a gelatin-like carbohydrate and a soluble fiber in the cell walls of plants, increased SCFA-producing species in the gut microbiota (e.g., *Bacteroides*), thereby increasing SCFA concentrations (e.g., acetic acid, propionic acid) and alle*via*ting the NAFLD phenotype (92). Shenling Baizhu powder, a formulation comprising a variety of natural medicinal plants, effectively improved NAFLD in high-fat–diet-fed rats by increasing the beneficial gut microbiota and reducing portal vein transport of LPS (166).

Maternal obesity and high-fat diet consumption both alter the gut microbiota of their offspring and increase their risk of developing NAFLD (168, 169). Therefore, interventions that target mothers are one approach for preventing NAFLD in children. In mice, early maternal diet intervention by restricting high fat consumption effectively reduced the incidence of NAFLD in the pups (170). Another murine study showed that short-term maternal treatment with a potent antioxidant, pyrroloquinoline quinone, prior to weaning attenuated disruptions in macrophage and microbiota function (134); these findings suggest that early reshaping of the gut microbiota combined with macrophage reprogramming during early weaning may alle*via*te the sustained proinflammatory environment, preventing the rapid progression of non-alcoholic fatty liver to NASH in the offspring of obese mothers.

Several pitfalls should be considered when translating the results of gut microbiome research from mouse models to humans. Two phyla, Bacteroidetes and Firmicutes, predominate in the gut microbiotas of both humans and mice (171). However, exploration at deeper taxonomic classifications revealed that 85% of the bacterial genera in the gut microbiota of mice are not present in human (172). Meta-analysis of datasets demonstrated that genera highly abundant in the human gut microbiota include *Prevotella, Faecalibacterium*, and *Ruminococcus*, whereas *Lactobacillus, Alistipes*, and *Turicibacter* are more abundant in the mouse gut microbiota; in contrast *Clostridium, Bacteroides*, and *Blautia* show similar relative abundance in both organisms (171). Therefore, results from animal experiments must be interpreted and applied carefully in gut microbiota-targeted translational research targeted toward the prevention and treatment of NAFLD.

## Conclusions

The etiologies of NAFLD in children and adolescents are multifactorial (e.g., high-fat diet, insufficient exercise, gender, maternal obesity, antibiotic use), but each of these factors impairs the gut microbiota community and leads to the dysbiosis. Dysbiosis promotes the development of NAFLD *via* the alteration of gut–liver homeostasis, including disruption of the gut barrier, portal transport of LPS to the liver, dysregulated BA profiles, and decreased concentrations of SCFAs. In terms of prevention and treatment, conventional approaches (e.g., dietary and exercise interventions) against obesity and NAFLD have been confirmed to recover dysbiosis of the gut microbiota and dysbiosis-mediated altered metabolism. Increased understanding of the importance of gut microbiota-mediated homeostasis in preventing NAFLD supports the potential usefulness of gut microbiota-targeted preventive and therapeutic strategies (e.g., probiotics) in children and adolescents. Continued research likely will disclose new gut microbiota genera and genes related to NAFLD and will also reveal the detailed mechanisms through which the gut microbiota influences the development of NAFLD. This information will be key to establishing safe and effective gut microbiota-targeted dietary management to combat NAFLD in children and adolescents.

## Author Contributions

The author confirms being the sole contributor of this work and has approved it for publication.

## Conflict of Interest

The author declares that the research was conducted in the absence of any commercial or financial relationships that could be construed as a potential conflict of interest.
